# RETRACTION: Efficacy of Anti‐Vascular Endothelial Growth Factor and Mitomycin C on Wound Gealing After Trabeculectomy in Glaucoma Patients: A Meta‐Analysis

**DOI:** 10.1111/iwj.70361

**Published:** 2025-03-18

## Abstract

**RETRACTION:** LiuW. and LiuB. , “Efficacy of Anti‐Vascular Endothelial Growth Factor and Mitomycin C on Wound Gealing After Trabeculectomy in Glaucoma Patients: A Meta‐Analysis,” International Wound Journal 21, no. 4 (2024): e14517, 10.1111/iwj.14517.38087907 PMC10961033

The above article, published online on 13 December 2023, in Wiley Online Library (http://onlinelibrary.wiley.com/), has been retracted by agreement between the journal Editor in Chief, Professor Keith Harding; and John Wiley & Sons Ltd. Following an investigation by the publisher, all parties have concluded that this article was accepted solely on the basis of a compromised peer review process. The editors have therefore decided to retract the article. The authors did not respond to our notice regarding the retraction.

**RETRACTION:** W. Liu and B. Liu , “Efficacy of Anti‐Vascular Endothelial Growth Factor and Mitomycin C on Wound Gealing After Trabeculectomy in Glaucoma Patients: A Meta‐Analysis,” International Wound Journal 21, no. 4 (2024): e14517, 10.1111/iwj.14517.38087907 PMC10961033

The above article, published online on 13 December 2023, in Wiley Online Library (http://onlinelibrary.wiley.com/), has been retracted by agreement between the journal Editor in Chief, Professor Keith Harding; and John Wiley & Sons Ltd. Following an investigation by the publisher, all parties have concluded that this article was accepted solely on the basis of a compromised peer review process. The editors have therefore decided to retract the article. The authors did not respond to our notice regarding the retraction.

